# The maximum evaporative potential of constant wear immersion suits influences the risk of excessive heat strain for helicopter aircrew

**DOI:** 10.1371/journal.pone.0196606

**Published:** 2018-05-03

**Authors:** Andrew P. Hunt

**Affiliations:** 1 School of Exercise and Nutrition Science, Queensland University of Technology, Brisbane, Australia; 2 Institute of Heath and Biomedical Innovation, Queensland University of Technology, Brisbane, Australia; 3 Physical Ergonomics Group, Land Human Systems, Land Division, Defence Science and Technology Group, Melbourne, Australia; US Army Research Institute of Environmental Medicine, UNITED STATES

## Abstract

The heat exchange properties of aircrew clothing including a Constant Wear Immersion Suit (CWIS), and the environmental conditions in which heat strain would impair operational performance, were investigated. The maximum evaporative potential (*i*_*m*_*/clo*) of six clothing ensembles (three with a flight suit (FLY) and three with a CWIS) of varying undergarment layers were measured with a heated sweating manikin. Biophysical modelling estimated the environmental conditions in which body core temperature would elevate above 38.0°C during routine flight. The *i*_*m*_*/clo* was reduced with additional undergarment layers, and was more restricted in CWIS compared to FLY ensembles. A significant linear relationship (*r*^2^ = 0.98, *P*<0.001) was observed between *i*_*m*_*/clo* and the highest wet-bulb globe temperature in which the flight scenario could be completed without body core temperature exceeding 38.0°C. These findings provide a valuable tool for clothing manufacturers and mission planners for the development and selection of CWIS’s for aircrew.

## Introduction

Sudden and unexpected immersion in cold water is a life-threatening situation that can cause cold shock and lead to debilitating hypothermia and drowning [1]. To prevent loss-of-life around cold water many authorities including the European Aviation Safety Agency [2, 3], Transport Canada [4], the Australian Maritime Safety Authority [5], and the International Organisation for Standardisation [6] stipulate protective clothing requirements for occupational groups (including offshore oil and gas, search and rescue, helicopter crew and passengers, and military operations) working in maritime environments. A Constant Wear Immersion Suit (CWIS) is routinely worn by aircrew during pre-flight and flight activities with the intention of protecting the wearer against cold shock and body heat loss in the event of accidental immersion in cold water [4, 5]. To maximise thermal insulation and therefore protection from the cold, CWIS’s are often impermeable to prevent water ingress into the suit, which would compromise its thermal insulation [7, 8]. Furthermore, additional clothing layers can be worn underneath the CWIS to enhance the insulation provided by the clothing system [9]. While increased insulation and reduced permeability are beneficial for conserving body heat in cold water, these factors also act to restrict body heat loss while in the aircraft. Consequently, the clothing that protects against cold stress while immersed in water can be responsible for elevating heat stress during flight [10].

Heat stress within aircraft is primarily influenced by the environmental conditions and protective clothing worn by the passengers and crew, and to a lesser extent their metabolic rate which is usually low while seated [11]. The environmental conditions within the cockpit are commonly higher than the conditions outside and can contribute significantly to the heat stress to which pilots and aircrew are exposed. During flight activities, Wet-Bulb Globe Temperature (WBGT) was 1–4°C higher inside the cockpit of a UH-60 helicopter compared to outside the aircraft [12]. Similarly, in the Bell 206 and 212 helicopters cockpit WBGT was on average 7.2°C higher after one hour of standby on the ground through the range of 13–31°C WBGT [13]. Also, in a Lynx helicopter, WBGT was observed to rise to 34°C, 6–8°C above the outside WBGT of 28.2°C [11]. Anecdotal evidence even reports that air temperature within helicopters flying over the Norwegian coast, where CWIS’s are a year-round requirement, can reach maximums of 40°C [14]. Compounding the issue of heat stress due to the environmental conditions, the necessity to wear a CWIS was the most commonly reported (70%) cause of heat strain among Royal Navy Helicopter Aircrew [11]. When wearing highly insulative clothing ensembles, research has recommended that cockpit temperatures should be maintained as low as 10–14°C to ensure thermal comfort and below 18°C to prevent physiological strain [15]. Therefore, these studies show that the environmental heat stress within helicopter cockpits can far exceed the level required to minimise heat strain in aircrew wearing CWIS.

Elevated heat stress and heat strain have important implications for aircrew performance and health. An increase in the number of pilot errors (fixed wing aircraft) has been reported during the summer months (approximately 30°C, 50–80% relative humidity, WBGT 29–34°C) compared to winter [16]. Furthermore, a significant increase in pilot errors (rotatory wing aircraft) was observed when temperature alone was above 30°C, which included navigational errors, equipment loss, crashes and near misses [17]. Elevated thermal strain has also been associated with an increase in body core temperature correlated with the number of incorrect reactions during simulated pilot tasks [18]. Aircrew report greater feelings of heat-related fatigue, and a reduction in alertness, contentment, and calmness when experiencing heat strain [19]. During simulated flight activities a reduction in self-rated performance quality was observed (being “reduced” or “dramatically reduced”) and performance effort was increased (rated as “moderate” to “considerable”) when skin temperature was elevated (>37.0°C) [20]. Flight performance as rated by an independent observer also deteriorates (more errors) with elevated heat strain [20]. Due to these adverse outcomes of heat stress and elevated heat strain in aircrew, it is imperative that the environmental conditions in the cockpit are considered when choosing to wear CWIS ensembles during flight.

Requirements to wear CWIS ensembles are dictated by sea temperature and the requirement to protect against cold stress. However, little guidance is available to indicate the environmental conditions in which CWIS may elevate the risks of heat stress during flight. Therefore the aim of this study was to assess the heat exchange properties of a range of aircrew clothing ensembles of increasing thermal insulation (with and without a CWIS), and to identify the environmental conditions in which heat strain would impair operational performance.

## Methods

### Clothing systems

Two types of Aircrew Protective Clothing Configurations (APCC) were evaluated in the present study, distinguished by the incorporation of either a two-piece flight suit (Flyers shirt and trousers, Australian Defence Apparel) including long sleeve shirt and trousers (FLY) or a Constant Wear Immersion Suit (CWIS). The CWIS was a one piece garment manufactured from a light-weight and waterproof laminated fabric comprising inherently fire resistant woven fabric and a micro-porous breathable laminate and a knitted aramid lining. Each of these APCC was assessed with three levels of undergarments (Table 1). Items common to both APCC’s included a helmet (HGU-56/P, Gentex, USA), armour and survival vests (Air Warrior, AWAE), nomex flight gloves (GS/FRP-2TA, Transaero, USA), and underwear (100% cotton briefs).

**Table 1:** The undergarments comprising the aircrew protective clothing configurations.

	FLY			CWIS		
Undergarment Level	1	2	3	1	2	3
*Socks Marino*	+	+		+	+	
*Socks ECW*			+			+
*Combat Boot (terra)*	+	+		+	+	
*Cold Weather Boot* ^*a*^			+			+
*T-shirt (100% cotton)*	+					
*Thermal Underwear* ^*b*^		+	+	+	+	+
*Neckwarmer* ^*c*^		+	+	+	+	+
*Coveralls–long sleeve & leg*			+		+	+
*Coveralls–short sleeve & leg*						+

+ indicates the item was included in the ensemble.

^a^ Zamberlan Civetta

^b^ long sleeve undershirt and drawers (Wilderness Wear Australia)

^c^(Chute, Icebreaker). ECW–Extreme Cold Weather.

### Heat exchange properties

Tests of the thermal resistance and evaporative resistance of the APCC’s were conducted in accordance with standard test procedures [21, 22] on a static heated sweating manikin (Newton model P-352, Thermetrics, USA) comprised of 26 independently heated zones. The manikin was suspended in a standing posture within a wind-booth (3.0 m length; 1.0 m width; 2.3 m height) to ensure a consistent air-flow was drawn past the manikin (front to back) by two fans (custom made to fit wind-booth) placed 2.0 m behind the manikin. Environmental parameters were measured 0.5 m in front of the manikin, with ambient temperature sensors (30K5A1B, Betatherm/MTNW, USA) at 0.7 and 1.4 m height, and relative humidity (HMP50U, Vaisala, USA) and wind-speed (TSI 8475–06, TSI Incorporated, USA) measured at 1.0 m height. Thermal resistance was measured in air temperatures which were at least 12°C below manikin skin temperature, to ensure a minimum power input of 20 W/m^2^ for every zone, and 50% relative humidity. Evaporative resistance was measured in isothermal conditions of 35±0.5°C air temperature and 40% relative humidity. The manikin’s skin was saturated with distilled water prior to commencing, and the flow rate of water to each zone was independently controlled throughout the test to ensure that saturation of the skin was maintained. All tests were conducted in triplicate and at each of three wind-speeds including 0.4, 1.12, and 2.2 m/s. Manikin skin temperature and heat flux were recorded at 1 min intervals and a 30 min period of data was utilised in analysis when the manikin’s skin temperature (35.0±0.2°C) and heat flux (±3%) had stabilised.

Dry thermal resistance (*R*_*t*_) was calculated from each manikin zone by:

$$R_{t}=\frac{\left(Ts-Ta\right)}{A/H}$$

where *R*_*t*_ = Thermal resistance (°C∙m^2^/W), Ts = manikin skin temperature, Ta = ambient temperature, and A = zone surface area (m^2^), and H = zone heat flux (W)

Evaporative resistance (*R*_*et*_) was calculated from each manikin zone by:

$$R_{et}=\frac{(Ps-Pa)*A}{\left[H-(Tskin-Tamb)*A/Rt\right]}$$

where *R*_*et*_ = evaporative resistance (kPa∙m^2^/W), Ps = water vapour pressure at manikin’s sweating surface (kPa), and Pa = ambient vapour pressure (kPa).

Total thermal resistance (*R*_*t*_) and evaporative resistance (*R*_*et*_) of the ensembles were calculated as a weighted average across all manikin zones using the parallel method [23, 24], whereby the area-weighted temperature of all manikin zones are summed and averaged, the heat flux to all zones are summed, and the areas are summed before total resistance is calculated [21, 22]. The average of the three tests was taken as the ensembles total thermal resistance and evaporative resistance. Total thermal insulation values were calculated by converting total thermal resistance to clo units (*I*_*t*_), as one clo is equivalent to 0.155 K∙m^2^/W [22]. A permeability index (*i*_*m*_) [25] was calculated by:

$$i_{m}=\frac{K*Rt}{Ret}/1000$$

where *i*_*m*_ = Permeability index (dimensionless), K = constant (60.6515 Pa/°C)

The ratio of the permeability index and insulation (*i*_*m*_*/clo*) was also calculated. The *i*_*m*_*/clo*, or the maximum evaporative potential, describes the fraction of maximum evaporative cooling that a wearer could achieve in a given environment [26].

A reference ensemble was assessed on the manikin prior to the test series to ensure the consistency of the test apparatus and procedure with international laboratories [21, 22]. The reference ensemble consisted of protective Nomex® long sleeve shirt and trousers, underwear, t-shirt, socks, and athletic shoes. Intrinsic thermal insulation (*R*_*cl*_) and evaporative resistance (*R*_*ecl*_) were calculated with a clothing area factor of 1.22 and the thermal insulation and evaporative resistance of the air layer around the nude manikin (*R*_*a*_). At 0.4 m/s wind-speed, *R*_*cl*_ was 0.118°C∙m^2^/W (0.76 clo), compared to the mean of 0.122°C·m^2^/W from the international community, which was within the 95% reproducibility limit of 0.024°C∙m^2^/W [21]. The *R*_*ecl*_ was 0.016 kPa∙m^2^/W, and within the 95% reproducibility limit of 0.008 kPa∙m^2^/W of the international community mean 0.016 kPa∙m^2^/W [22]. These data support the validity and reliability of the test procedures as they are comparable to the international community.

### Biophysical modelling of heat strain

Body core temperature elevation and duration limits for flight activities were determined based on a previously validated biophysical model [27]. Further details on biophysical modelling techniques can also be found in several recent reviews [28, 29]. Model calculations were performed based on an average male, 180 cm in height, a body mass of 80 kg, 14% body fat, and on the assumptions that the individual was healthy, acclimatised, hydrated and well-rested. Commencing with a body core temperature of 36.9°C, the model calculations were performed in a range of environmental conditions (Table 2), with wind-speed assumed to be at a constant 0.2 m/s. The flight scenario was based on a typical reconnaissance sortie and included a pre-flight period and a flight period [30, 31]. Although the durations of these periods will vary for each specific mission and circumstance, for the purpose of this modelling evaluation the pre-flight check of the aircraft was set to 20 minutes and the flight period was set to 240 minutes (4 hours). Metabolic rates were selected to represent typical work rates during the pre-flight and flight periods. Research has shown the work rate of rotary-wing aircraft pilots to be of a low intensity during flight, ranging between 100–240 W [11, 32, 33]. The pre-flight activities are generally of a moderate work intensity, with metabolic rates up to 206–490 W [11, 32]. Therefore metabolic rates included in the modelling were 350 W for pre-flight activities and 150 W during flight. The protective clothing inputs to the modelling included each ensembles total thermal insulation and evaporative resistance. For the flight period, the effects of transitioning from a standing to a seated posture on the clothing heat exchange properties was accounted for by correcting the heat exchange properties by an established relationship between standing and seated postures [34].

**Table 2:** The range of environmental conditions in the biophysical modelling.

Dry Bulb Temperature (°C)	Globe Temperature (°C)	Wet Bulb Temperature (°C)	Relative Humidity (%)	WBGT (°C) *
20	30	14	50	18
21	31	15	52	19
22	32	16	53	20
23	33	17	54	21
24	34	18	55	22
26	36	20	57	24
27	37	21	58	25
28	38	22	58	26
30	40	24	60	28
32	42	26	62	30
34	44	28	63	32
36	46	30	64	34
38	48	32	65	36

* Wet-Bulb Globe Temperature was calculated as: WBGT (°C) = 0.7*T_wet_ + 0.2*T_globe_ + 0.1*T_dry_.

### Analysis

To guide the risk management procedures when flying under conditions of high heat stress the modelling analysis was conducted up until a body core temperature of 38.0°C was reached. This level of thermal strain is commensurate with industry and population guidelines for work in hot environments [35–37]. Working for longer durations would foreseeably increase the risk of heat-related illness and injury and may predispose the individual to cognitive deficits which would impair flight performance.

To examine the association between environmental conditions and heat strain when wearing the APCC’s, the relationship between *i*_*m*_*/clo* and WBGT was examined. The *i*_*m*_*/clo* for each ensemble was compared to the highest WBGT in which body core temperature could stabilise below 38.0°C. Linear regression was performed to evaluate the strength of the relationship, with statistical significant accepted at α<0.05.

## Results

### Heat exchange properties of the aircrew protective clothing configurations

**Fig 1:**
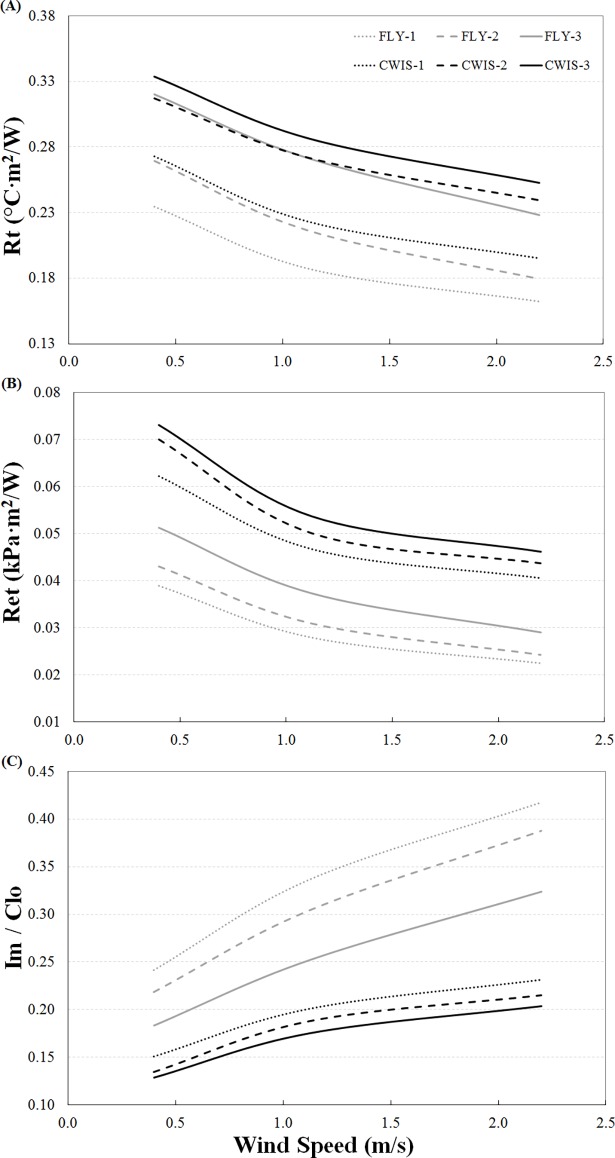
Total thermal insulation (A), total evaporative resistance (B), and maximum evaporative potential (C) of the APCC across three wind-speeds.

Total thermal insulation (*R*_*t*_) for both the FLY and CWIS configurations increased with additional undergarments (Fig 1A). FLY-1 provided the least and CWIS-3 the most insulation of all the APCC. There was an overlap in the insulation provided by the FLY and CWIS configurations, such that FLY-2 had a similar insulation to CWIS-1, and FLY-3 had a similar insulation to CWIS-2. Elevations in wind-speed reduced the *R*_*t*_ provided by each of the APCC’s.

Total evaporative resistance (*R*_*et*_) of the APCC’s increased with the inclusion of undergarment layers in the ensemble (Fig 1B). However, there was no overlap in evaporative resistance between the FLY and CWIS, with the CWIS ensembles possessing a distinctly higher *R*_*et*_ compared to the FLY configuration. *R*_*et*_ was reduced as wind-speed rose to 1.12 m/s, and to a lesser extent up to 2.2 m/s; this trend being observed similarly for all APCC’s.

The maximum evaporative potential (*i*_*m*_*/clo*) was consistently reduced with additional undergarment layers, and restricted to a greater extent in the CWIS compared to the FLY ensembles (Fig 1C). The improvement in maximum evaporative potential with increasing wind-speed was diminished in the CWIS, particularly at the higher wind-speed, compared to the FLY ensembles.

### Biophysical modelling of heat strain

**Fig 2:**
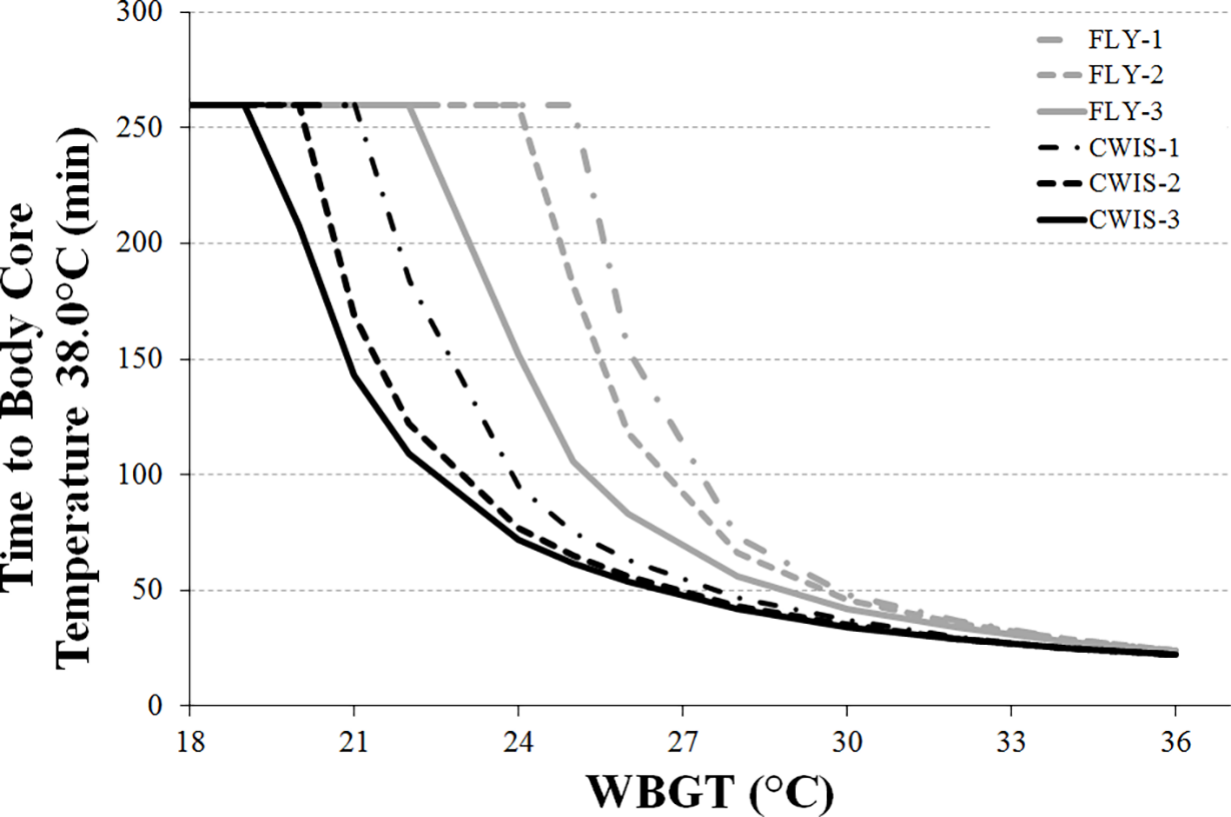
The time until a body core temperature of 38.0°C across a range of WBGT for each of the APCC’s. (Note: 260 min was the maximum duration modelled, including 20 min pre-flight time and up to 240 min flight time).

Flight duration, before body core temperature reached 38.0°C, was progressively shortened with both APCC undergarment levels and rising WBGT (Fig 2). The CWIS configurations showed much quicker elevations in body core temperature in the WBGT range of 19–22°C compared to the FLY ensembles. Consequently the flight durations were more restrictive in the CWIS configurations. Alternatively, in the most oppressive conditions (WBGT >30°C) the differences in flight duration became narrower between the FLY and CWIS ensembles.

**Fig 3:**
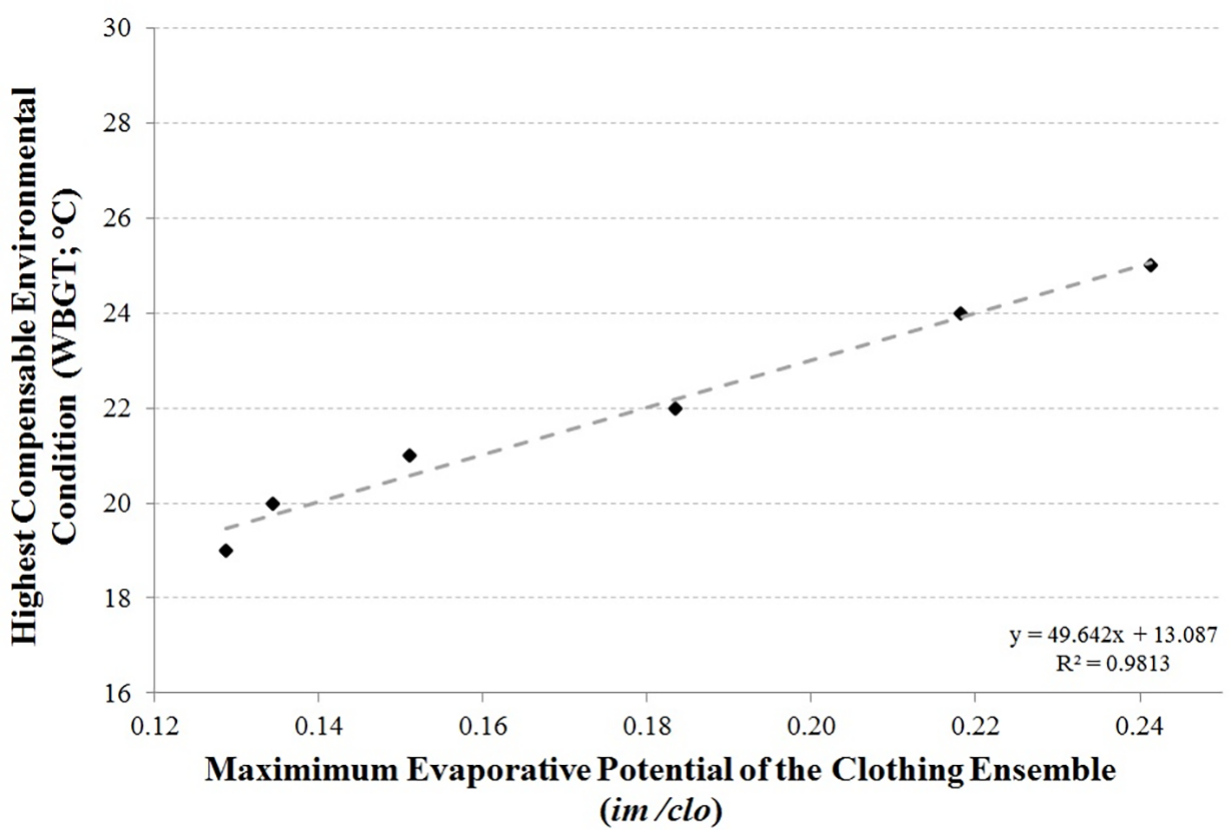
The relationship between maximum evaporative potential (*i*_*m*_*/clo)* and the highest compensable environmental conditions (WBGT) in which the flight scenario could be completed without body core temperature exceeding 38.0°C.

APCC’s that were more restrictive to heat exchange (lower *i*_*m*_*/clo*) required cooler environmental conditions (lower WBGT) in order to prevent elevations in body core temperature above 38.0°C. A significant linear function (*r*^*2*^ = 0.98, *P*<0.001) represented the relationship between *i*_*m*_*/clo* and the highest WBGT in which the flight scenario could be completed without body core temperature exceeding 38.0°C (Fig 3). The WBGT at which body core temperature progressed above 38.0°C was consistently higher for the CWIS compared to the FLY ensembles, and as the level of undergarments increased within the two APCC types.

## Discussion

The present study demonstrates that the incorporation of a CWIS into aircrew protective clothing systems restricts evaporative heat loss and elevates heat strain in aircrew during flight activities. The maximal evaporative potential (*i*_*m*_*/clo*) of the APCC’s was found to be closely related to the environmental conditions (WBGT) in which body core temperature could stabilise below 38.0°C during a routine flight scenario. Furthermore, in environmental conditions above this WBGT limit, the flight duration before reaching a body core temperature of 38.0°C became progressively restricted. Therefore, the findings of the present study provide a valuable tool for evaluating the risk of elevated heat strain when aircrew wear CWIS ensembles, and guidance on restrictions to flight duration when CWIS ensembles are worn in environmental conditions that exceed the tolerable WBGT limits.

### Heat exchange properties of the APCC

Helicopter aircrew wearing survival suits, even in relatively cool conditions (18°C), note feelings of thermal discomfort due to raised skin temperature and sweat accumulation in the clothing layers [38]. Indeed, the necessity to wear a CWIS was the most commonly reported (70%) cause of heat strain among Royal Navy Helicopter Aircrew [11]. In support of these observations with aircrew, the heat exchange properties of the APCC’s measured in the present study, particularly the total evaporative resistance (Fig 1), suggest that wearing the CWIS configurations would cause greater thermal discomfort for aircrew compared to the FLY configurations. Thermal discomfort in warm environments is closely correlated with whole body (and local) skin wettedness (the amount of moisture on the skin) [39]. During work in the heat where sweat is produced, there would likely be a higher water vapour pressure within the microclimate of the CWIS, compared to the FLY configurations. This is due to the higher resistance of water vapour transfer through the immersion suit, which reduces sweat evaporation from the skin and increases skin wettedness. In addition, skin wettedness affects the interaction between the skin and fabric, with the coefficient of friction (resistance to movement of the fabric over the surface of the skin) increasing with skin wettedness above 25% [40]. Consequently, the texture of a fabric is perceived to be “rougher” and less pleasant with increases in skin wettedness. Therefore, aircrew wearing the CWIS configurations are likely to have higher skin wettedness contributing to greater thermal discomfort and material discomfort. These clothing properties are also likely to be detrimental to aircrew performance and heat strain.

### Preventing excessive heat strain

Excessive heat stress has the potential to be detrimental to the performance and health of aircrew. Flight performance can be impaired when heat stress is elevated, as evidenced by an increase in the number of pilot errors [16, 17]. Furthermore, elevated heat strain has been associated with an individual’s perception of their own task performance, and flight performance as rated by an independent observer, when wearing CWIS. Individuals wearing CWIS have reported greater feelings of heat-related fatigue, which was determined by reduction in alertness, contentment, and calmness ratings [19]. Similarly, during simulated flight activities a reduction in self-rated performance quality and increased performance effort has been observed when skin temperature was elevated (>37.0°C) [20]. The increase in body core temperature has been also correlated with the number of incorrect reactions during simulated pilot tasks [18]. Consequently, the elevated heat strain caused by wearing the CWIS ensembles observed in the present study is likely to impair performance of critical flight tasks as environmental conditions become warmer.

The present findings demonstrate two important outcomes in order to prevent excessive heat strain in aircrew wearing CWIS. Firstly, the maximal evaporative potential of APCC’s is significantly correlated to the environmental conditions in which body core temperature does not exceed 38.0°C. Aircrew protective clothing with lower maximum evaporative potential require cooler environmental conditions to prevent excessive heat strain during flight scenarios (Fig 3). For the most restrictive ensembles (CWIS-3) the environmental conditions required to prevent excessive heat strain were below a WBGT of 19°C. Similarly, previous findings indicated that physiological strain will progressively develop above 18°C when wearing aircrew clothing and a survival suit [15]. Building upon this work that has only assessed the most insulative CWIS ensembles, the present findings demonstrate the environmental limits for a range of APCC’s, with *i*_*m*_*/clo* in the range of 0.13 to 0.24 (Fig 3). Over this range there is a significant linear relationship to the highest WBGT in which body core temperature would not exceed 38.0°C, during the flight scenario assessed in the present study. This relationship could be utilised by clothing manufacturers and mission planners alike. For manufacturers, knowledge of the *i*_*m*_*/clo* of their clothing ensembles will aid in the development of clothing systems that will be suitable for the environmental conditions in which they are likely to be used by clientele. For mission planners, the relationship will enable an informed decision regarding the choice of APCC with which to equip their aircrew. A decision that was once primarily based on sea temperatures and survival time requirements can now be balanced with the risk of elevated heat strain during flight.

A second key finding highlights that mission planners need to give careful consideration to the type of clothing worn by aircrew across the range of WBGT evaluated in the present study. The findings demonstrate that progressive restrictions to flight duration would be required to prevent excessive heat strain as the environmental conditions (WBGT) become warmer (Fig 2). In a WBGT range above 22°C, ensembles incorporating a CWIS show a considerable reduction in flight duration before excessive heat strain develops compared to ensembles based on a regular flight suit. Above 25°C WBGT, there are marked reductions in flight duration wearing the FLY ensembles, while durations continue to decline for the CWIS ensembles albeit at a slower rate. Above 30°C WBGT, severe restrictions to flight duration are experienced, irrespective of the clothing system worn. These conditions reflect that the environment is now the primary limiting factor to body heat loss, rather than the protective clothing per se. Utilising these limitations to flight duration will provide a valuable tool for mission planners as they assess the clothing requirements to achieve the desired objectives, without placing aircrew at risk of excessive heat strain during flight.

Overall, the benefit of wearing a flight suit ensemble (e.g. FLY-1) is longer flight durations before an excessive elevation in heat strain. However, this comes with the risk of a short survival time if immersed in cold water [41]. Alternatively, an ensemble with a high insulation (e.g. CWIS-3) that will prolong survival time in cold water may also reduce flight duration due to elevated thermal strain. Therefore, the choice of protective clothing configuration to wear for a flight will be dependent on a number of factors [42]. These will include the objective of the mission and the time required to achieve that objective. This will coincide with the environmental conditions and the length of time before excessive heat strain may develop. The choice of clothing configuration to meet these mission specific requirements needs to be balanced with the risk of immersion in cold water. The choice of clothing configuration to protect against cold water immersion needs to consider the sea temperature and conditions (sea states) as well as the expected survival time, the duration of a rescue flight, and the effects of cold shock. These factors should be assessed on a case-by-case basis specific to each flight in order to balance the risks of excessive heat strain with survival time in cold water.

### Limitations

The recommendations of the present study are primarily focused on the flight scenario assessed, which included a 20 min pre-flight period of moderate intensity work (350 Watts) and up to 240 min flight period of low intensity work (150 Watts). These assumptions reflect the likely work intensity and metabolic rates of aircrew performing their routine duties [11, 30–32]. However it needs to be acknowledged that deviations from these assumptions, such as longer pre-flight checks or greater metabolic rates during flight, would likely cause greater heat strain than the findings presented here. In addition, individual variations such as resting body core temperature, hydration status, acclimatisation status, and levels of fatigue will also influence a persons’ tolerance to heat strain. These factors need to be considered in the implementation of risk management strategies to prevent excessive heat strain.

## Conclusion

The incorporation of a constant wear immersion suit into aircrew protective clothing configurations reduced the maximal evaporative potential of the clothing ensembles. Maximum evaporative potential was found to be linearly related to the environmental conditions (WBGT) in which body core temperature would not exceed 38.0°C during a routine flight scenario. In the event that environmental conditions exceed these limits, restrictions to flight duration would be required to ensure excessive heat strain is prevented. These findings provide a valuable tool for clothing manufacturers and mission planners for the development and selection of CWIS’s for aircrew.

## Supporting information

S1 Raw Data The data from thermal manikin testing are provided in the supporting excel spreadsheet. (XLSX)
